# In people with nociplastic dominant chronic pain is multi-disciplinary pain management plus motivational interviewing more effective than multi-disciplinary pain management alone for improving pain self-efficacy and pain interference: A randomised controlled trial protocol

**DOI:** 10.1016/j.conctc.2026.101697

**Published:** 2026-10-06

**Authors:** Jon Ford, Paul O'Halloran, Nicholas F. Taylor, Shane Pritchard, Andrew Hahne

**Affiliations:** a School of Allied Health, Human Services and Sport, La Trobe University, Melbourne, Australia; b Advance Healthcare, 157 Scoresby Road, Boronia, Victoria, 3155, Australia

**Keywords:** Chronic pain, Randomised controlled trial, Motivational interviewing

## Abstract

**Background:**

Chronic pain is the leading cause of disability and disease burden globally. Emerging evidence suggests that people with nociplastic dominant chronic pain commonly have heightened pain sensitivity, high levels of pain interference and low self-efficacy. There is low certainty evidence supporting the effectiveness of motivational interviewing in a range of health conditions including chronic pain. However, the impact of motivational interviewing as an adjunct to evidence-based multi-disciplinary pain management has not been adequately evaluated. The aim of this paper is to describe the protocol for a randomised controlled trial comparing multi-disciplinary pain management plus motivational interviewing to multi-disciplinary pain management alone for improving pain self-efficacy and pain interference in people with nociplastic dominant chronic pain.

**Methods:**

A multi-centre parallel group randomised controlled trial is proposed. 184 participants with nociplastic dominant chronic musculoskeletal pain referred for multi-disciplinary pain management will be recruited. All participants will receive multi-disciplinary pain management over 12 weeks with one group having adjunctive motivational interviewing. Outcomes will be assessed at 12, 26 and 52 weeks following randomisation. The co-primary outcomes will be the Pain Self-Efficacy Questionnaire and Brief Pain Inventory pain interference scores at 52 weeks. Data will be analysed according to intention-to-treat principles and estimate between-group effects using linear mixed models.

**Conclusion:**

This trial will determine the difference in outcomes between multi-disciplinary pain management plus motivational interviewing and multi-disciplinary pain management alone for chronic nociplastic dominant pain.

Chronic pain is the leading cause of disability and disease burden globally [1,2]. Approximately 20% of people over 45 years of age have chronic pain leading to activity limitation, care seeking, opioid use and hospitalisation [3].

Multi-disciplinary pain management has demonstrated effectiveness in chronic pain, however the clinical importance of the observed effects is questionable [4]. It has been hypothesised that treatment individualised to the type of pain problem rather than a “one size fits all” approach could demonstrate larger effects [5]. Specifically, people with nociplastic dominant pain and probable mechanisms of heightened pain sensitivity may be more responsive to interventions focusing on time contingent graded activity/exercise that address the psychosocial barriers to recovery [6,7].

Nociplastic pain can be defined as “pain that arises from altered nociception despite no clear evidence of actual or threatened tissue damage causing the activation of peripheral nociceptors or evidence for disease or lesion of the somatosensory system causing the pain”(8). There is emerging consensus on the clinical features and mechanisms underpinning nociplastic dominant pain although further research is recommended before clinical implementation [7,[8], [9], [10]].

Self‐efficacy can be defined as an efficacy expectation where a person believes that taking a particular action will result in a specific and positive outcome [11]. Improving pain-related self-efficacy is associated with improvements in pain, pain interference and a range of other psychosocial outcomes relevant to nociplastic dominant pain [[12], [13], [14]]. Treatment for chronic pain aimed at improving self-efficacy has therefore been suggested as being important [15] and may contrast to traditional or “passive” methods of delivering health related education/information that may not enhance self-efficacy or optimise outcomes [15,16] including adherence [17]. However the association between self-efficacy, adherence and outcomes in people with chronic pain has not been established [18].

One method with potential to improve self-efficacy and other clinical outcomes in people with nociplastic dominant pain is motivational interviewing (MI). Motivational interviewing is a person centred collaborative conversation style for strengthening a person's own motivation and commitment to change that has variable evidence of effectiveness in chronic pain [[19], [20], [21]] as well as other chronic health conditions [[22], [23], [24], [25]]. In order to be classified as motivational interviewing, practitioners need to adhere to a spirit that emphasises partnership, acceptance of the client, evocation and compassion. Given the emphasis on engagement with patients and resolving ambivalence to change, MI may be a useful adjunctive treatment for improving therapeutic alliance and barriers to engaging in graded activity/exercise, adherence and clinical outcomes [[26], [27], [28], [29], [30]]. However, MI effectiveness may vary across providers, populations, target problems, settings and whether it is delivered as intended (i.e. treatment fidelity) [31].

A recent perspective paper suggested that MI and education based on pain management principles are complementary and likely to result in larger treatment effects for people with chronic pain [32]. One recent cluster randomised controlled trial evaluating MI as an adjunctive treatment to multi-disciplinary pain management showed modest improvements in return to work but not pain or disability [33]. In keeping with much of the MI literature [31], the treatment protocol was not clearly defined and insufficient treatment fidelity measures were reported [34]. This is important because multiple systematic reviews have shown larger treatment effects with MI in clinical trials when fidelity has been demonstrated [35,36].

Given the burden of chronic pain to society and individuals and the potential to improve outcomes through adjunctive treatment that addresses self-efficacy, a randomised controlled trial is planned evaluating the effectiveness of multi-disciplinary pain management plus motivational interviewing (PM + MI) compared to multi-disciplinary pain management alone (PM) for improving pain self-efficacy and pain interference in people with nociplastic dominant chronic pain. It is hypothesised that adding motivational interviewing to standard pain management will result in greater long-term (52 week) improvement in self-efficacy and pain interference.

## 1 Methods

### 1.1 Study design

This will be a parallel group randomised controlled trial comparing PM to PM + MI (Fig. 1). All participants will receive a standard multi-disciplinary pain management program delivered by physiotherapists, psychologists and medical practitioners working at one of five tertiary care practices in metropolitan Melbourne, Australia (Advance Healthcare). One group will also receive the adjunctive treatment of MI provided by one of the trial physiotherapists, nested within the pain management program (Fig. 1).

**Fig. 1:**
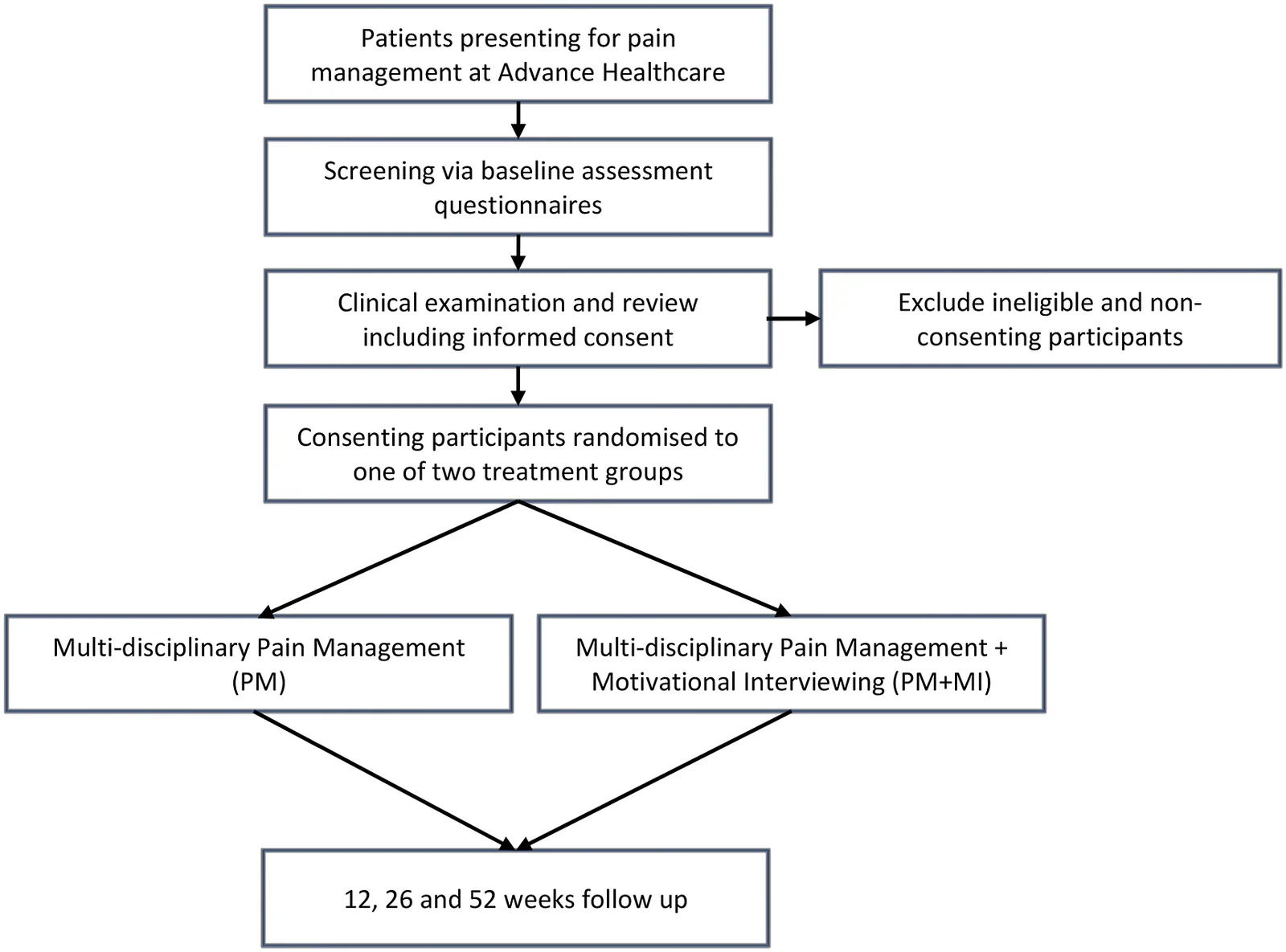
Study design.

### 1.2 Ethics and registration

Research ethics has been approved by the La Trobe University Human Ethics Committee (Ethics ID #HEC21026). The trial is prospectively registered with the Australia & New Zealand Clinical Trials Registry (ACTRN12622000829718).

### 1.3 Patient and public involvement

There was no formal patient or public involvement in the design of the trial.

### 1.4 Recruitment

Patients presenting for Pain Management at any of the five participating centres will be informed of the trial following standard assessment, and offered the opportunity to consent to participate if they meet the eligibility and screening criteria.

### 1.5 Eligibility and screening

People referred for multi-disciplinary pain management are routinely assessed by one of the physiotherapists. Assessment consists of a complete history and physical examination and a series of self-administered questionnaires.

On completion of this assessment, physiotherapists will discuss participation in the trial if participants meet the following inclusion criteria:

i. Have a primary complaint of chronic musculoskeletal pain (of greater than 3 months duration);
ii. Are presumed to have a pain type of nociplastic dominant based on the pain management team's collective judgement including the use of the best available clinical criteria (Table 1) [7,10];

**Table 1:** Clinical criteria and grading for nociplastic pain affecting the musculoskeletal system [7].

1. The pain is: • Chronic (>3 months) • Regional (rather than localised and/or neuroanatomically plausible) in distribution • There is insufficient evidence that nociceptive pain (a) is present or (b) if present, is the dominant pain type • There is insufficient evidence that neuropathic pain (a) is present or (b) if present, is the dominant pain type

2. There is a history of pain hypersensitivity in the region of pain. Any one of the following: • Sensitivity to touch • Sensitivity to pressure • Sensitivity to movement • Sensitivity to heat or cold

3. Presence of comorbidities. Any one of the following: • Increased sensitivity to sound and/or light and/or odours • Sleep disturbance with frequent nocturnal awakenings • Fatigue • Cognitive problems such as difficulty to focus attention, memory disturbances, etc

4. Evoked pain hypersensitivity phenomena can be elicited clinically in the region of pain. Any one of the following: • Static mechanical allodynia • Dynamic mechanical allodynia • Heat or cold sensitivity • Painful after-sensations reported following the assessment of any of the above alternatives

Possible nociplastic pain: 1 and 4. Probable nociplastic pain: all the above [1,2,3,and4]]
iii. Have been referred for a multi-disciplinary pain management program;
iv. Are aged between 18 and 75 years;
v. Have sufficient fluency in English to complete all baseline and follow up questionnaires;
vi. Have a Pain Disability Questionnaire score ≥ 50/150 and a Numerical Pain Rating Scale score ≥ 3/10, to ensure that participants had sufficiently high pain and disability to be representative of those typically presenting to Multi-disciplinary Pain Management.

Participants will not be approached for consent if any of the following exclusion criteria are met, but reasons for exclusion will be recorded:

i. Red flag pathologies unsuitable for pain management (active cancer, signs of cauda equina syndrome based on bladder or bowel disturbance, risk of spinal fracture, signs of potential infection, systemic inflammatory disease);
ii. Chronic cancer, pelvic or neuropathic (eg postherpetic neuralgia) pain;
iii. Recent or pending surgery for the pain condition (9 months prior or in the next 9 months);
iv. Pregnancy or childbirth within the last 6 months;
v. Injections for the pain problem within the last 6 weeks, as we wish to study treatment effects independent of the effects of such procedures;
vi. Have already received multi-disciplinary pain management for the main pain problem within the last 2 years, to avoid contamination.

Definitive diagnostic accuracy recommendations for nociplastic dominant pain type are not available and therefore it is unlikely that a suitable reference standard will be developed. However, individualising treatment based on pain type is recommended by expert panels on chronic pain [7] and preliminary validation data [10] and is particularly relevant when determining the optimal behavioural approach most appropriate [5].

### 1.6 Randomisation and concealed allocation

A randomisation schedule for allocating participants to either PM or PM + MI will be prepared in advance by an offsite researcher not involved in assessing, enrolling or treating the participants. Block randomisation (random block lengths), with stratification for treatment centre (5 levels) and primary pain site (5 levels: low back, neck, upper limb, lower limb, widespread) will be employed. To enrol a participant, the treatment centre will email the consenting participant's name and date of birth to an offsite randomisation service who will allocate the participant to a treatment group in accordance with the randomisation schedule.

### 1.7 Blinding

Participants will be blinded to the study hypothesis and they will not be told what treatments are being compared in the trial. They will all be informed that they will receive a standard pain management program, but that some participants will receive additional elements to this that they are unlikely to notice. Grouped data without identifying group labels will be provided to the statistician for analysis. It is not possible to blind treating physiotherapists, as the clinical notes will indicate to which group the patients have been allocated. The statistician will receive grouped data without identifying treatment labels.

### 1.8 Treatment

The pain management program for which the participant had been referred will not change based on participation in the study for the PM group. Treatment will consist of physiotherapy, psychology, medical ± dietitian individual ± group sessions over a 12-week period attending 2-3 times per week. Components of the treatment will be consistent with recommended standards for multi-disciplinary pain clinics [37,38]. A description of the experimental and comparison group treatments is provided in Table 2. Advance Healthcare will provide facilities, personnel and resources so that trial treatment is provided at not cost to the participant.

**Table 2:** Description of experimental and comparison group treatments according to the template for intervention description and replication (TIDieR) [39].

	Pain management + motivational interviewing group	Pain management group
Brief name	Pain management + motivational interviewing (PM + MI)	Pain management (PM)
Why	Improve pain and activity limitation by way of greater self-efficacy in pain management participants, with better maintenance of treatment effects in the long-term	Improve quality of life through a goal oriented graded activity/exercise program
What materials	Practitioner delivering the treatment maintains detailed clinical notes to guide and record the content of each session and deviations from protocol	Pain management team maintains detailed clinical notes on multi-disciplinary treatment provided
What procedures	To adhere to the motivational interviewing spirit when delivering MI micro-skills during processes of engagement, focusing, evoking and planning to increase goal oriented graded activity/exercise as well as address specific barriers to recovery	Assess the barriers to recovery and provide a 12-week individual and group pain management program
Who provided	Trial physiotherapists who have received training in motivational interviewing by attendance at 10 h of group workshop training and one on one coaching from an expert motivational interviewing practitioner	Medical, physiotherapy (different from the PM + MI physiotherapists), psychology and dietitian practitioners with a special interest in chronic pain
How provided	Individual and group sessions face to face or videoconferencing/telephone if requested	Individual face to face or videoconferencing if required
Where (setting)	Participant with chronic pain living independently in the community attending one of 5 local clinics	Participant with chronic pain living independently in the community attending one of 5 local clinics
When/how much (dose)	10 dedicated motivational interviewing sessions each of 45 min duration over 40 weeks (total 7.5 h) incorporated into the standard multi-disciplinary pain management program (no additional sessions)	12 weekly individual psychology sessions, 16 group psychology sessions (2/week), 32 individual physiotherapy sessions (3/week), 4 group dietitian sessions ± 4 individual dietitian sessions (if indicated), >4 individual medical sessions over 12 weeks with low frequency physiotherapy ± psychology/medical sessions between discharge and 52 weeks
Tailoring	Sessions tailored to the needs and progress of the individual	Sessions tailored to the needs and progress of the individual
Fidelity checking measures	Physiotherapy adherence to core MI skills and spirit as assessed by the Motivational Interviewing Treatment Integrity code (MITI) on an audiotaped session Treatment delivery through number of completed sessions and treatment descriptions from physiotherapy clinical notes Treatment receipt and enactment by the participant through exit interviews on a proportion of the sample and goal sheet analysis on all participants.	Physiotherapy skills and treatment delivery assessed with Motivational Interviewing Treatment Integrity code (MITI) on a sample of audiotaped sessions for evidence of core MI skills and spirit Treatment delivery through number of completed sessions and treatment descriptions from physiotherapy clinical notes Treatment receipt and enactment by the participant through exit interviews on a proportion of the sample and goal and rehabilitation sheet analysis on all participants.

### 1.9 MI treatment protocol

Participants in the PM + MI group will receive the same multi-disciplinary pain management program with the physiotherapist providing adjunctive MI treatment within this program. This will involve integration of MI principles into the routine conversations taking place throughout the pain management program consultations. There will be ten 45-min MI-specific sessions with pre-determined foci commencing early in the program and continuing until the final session 24 weeks after discharge. This will enable more in-depth application of micro-skills and processes of Motivational Interviewing. The 10 MI specific sessions will replace physiotherapy sessions in the standard pain management program, so both groups will have an equal number of sessions.

The treatment protocol was developed by the research team from existing literature and the training expertise of the research team (a combined total of over 25 years of MI training experience that includes membership of the Motivational Interviewing Network of Trainers). The protocol aims to facilitate an increase in goal oriented activity/exercise as well as behaviour that addresses barriers to recovery. Physiotherapists will use MI communication skills consistent with the spirit of MI. The aim will be to elicit responses that are consistent with change (referred to as change talk) that predict increased motivation to change [40]. These skills will include open questions, affirmations, reflections and summarising (Table 3).

**Table 3:** Motivational interviewing skills [40].

Motivational interviewing skills	Embedding within all physiotherapy consultations
Open questions (O): Questions that cannot be answered with a yes/no response	Asking open questions to elicit the participant's perspective in a manner that demonstrates empathy/understanding and optimises engagement/change talk. An example of an open question is “What do you see as the major benefits of being more active?”
Affirmations (A): Positive comments on the participant's efforts	Identifying positive past performances in implementing change (no matter how small) to optimise engagement/change talk and build confidence to change. An example of an affirmation in a participant who continued to exercise despite feeling quite down during the week is “You were still able to continue with your exercise even when you were feeling uncomfortable. That shows great determination and effort”.
Reflections (R): The physiotherapist's attempt to understand what they are hearing from the participant *Simple:* Repeat or slightly rephrase what has been said *Complex:* Goes beyond a simple reflection in that we reflect what we understood was meant (can involve taking an informed guess)	Reflecting key components of the participant's language to demonstrate empathy/understanding and foster change talk. An example of a complex reflection during a supervised exercise session is when a patient comments that the exercise is hard “In a good way” the physiotherapist then reflects to the patient “It feels good to be moving again”.
Summaries (S): A type of reflection that summarises the conversation key points that are likely to facilitate change in the participant. Are useful for bringing important information together, moving forward or providing an opportunity for patients to correct misunderstandings	Summarising a participant's core points related to change in activity/exercise. For example, therapists are taught how to take a brief moment at the start of the consultation to summarise progress over the last week or clarify any issues or to summarise what the client has committed to do at the end of the session.

Physiotherapists will structure the MI intervention around the processes of engagement (practitioner and participant working together through open questions or reflections demonstrating empathy and understanding), focusing (setting the agenda on what to change), evoking reasons for change and change talk (through open questions and reflections) and planning how to change (with a focus on the participant directing the planning process).

Using these skills and processes, participant change talk regarding the reasons, needs and desires to increase their level of activity/exercise will be elicited. Participant-rated importance, confidence and commitment to change will also be facilitated, in part by eliciting, reflecting on and affirming positive past performances in implementing change. Information will be provided in an MI consistent way, typically towards the planning stage, and always while providing the participant an opportunity to review this information within the context of their own perspective.

Training will be oriented to equip the physiotherapists with the knowledge and skills to demonstrate competence on the Motivational Interviewing Treatment Integrity Code (MITI 4.2.1) [41]. This coding system evaluates the competency of a practitioner based on the component MI processes using global scores and specific behaviour counts demonstrated by the practitioner [4].

Engagement is the typical focus of early communications in MI and this will in part be facilitated by the initial treatment sessions conducted by the physiotherapist. Three MI specific sessions will be conducted early in the pain management program (weeks 1, 2 and 4), with the goal of eliciting and strengthening (in a non-threatening manner) an understanding of MI principles, the participant's own values based goals, behaviours that address specific barriers to recovery and readiness to undertake goal oriented graded activity/exercise. In the early to mid-stages of the pain management program, the participant will commence a home based graded activity and clinic based supervised exercise program. Four MI specific sessions (weeks 5, 7, 9 and 12) will be conducted reviewing progress with this program and using MI communication skills and principles to elicit and strengthen graded progressions in activity/exercise. The sessions will also explore any challenges/barriers with the graded activity/exercise program and consider medium to longer term goals including return to work. Following discharge from the program three MI specific sessions (4, 11 and 24 weeks after discharge) will be conducted with the aim of supporting the ongoing goal oriented graded activity/exercise using MI skills and principles. This will be an important phase to optimise transition from the intensive pain management program to ongoing and relatively independent self-management.

### 1.10 Participating physiotherapists and treatment fidelity

Physiotherapists will complete a training program to enhance their skills in MI, developed by the research team and implemented by one of the authors (PO) before commencement of the trial comprising:

• Ten hours of group training sessions covering core MI content and specific training in how to embed MI into conversations during regular treatment and the ten MI specific sessions
• At least 6 face-to-face or telephone, one on one, coaching sessions, consisting of a 20-min role play with 10 min of feedback. In role plays, the trainer assumes the role of a participant with an actual behavioural change they have been considering implementing as distinct from a role play that is fictional
  • In the final session, the practitioner's competency in the delivery of MI is assessed using the Motivational Interviewing Treatment Integrity code (MITI 4.2.1) [42]. An external assessor not otherwise associated with the study, will review and score the role play, with at least fair competency and proficiency thresholds needing to be met (Table 4) before providing treatment in the trial. The assessor is a MINT Certified Trainer and currently serves as a Director Emeritus from the MINT Board of Directors focusing on professional skill development for MI practitioners and trainers. A pass standard (at least “fair” on technical/relational) on the MITI will be a requirement to commence treatment in the trial. Further training including feedback on the MITI results, will be provided if required until a pass standard is attained

**Table 4:** Description and scoring of the MITI codes [41,42].

MITI code	Brief description
Globals [[1], [2], [3], [4], [5]]	
Cultivating Change Talk (CC)	Encourages the client's own language in favour of the change goal and confidence for making that change.
Softening Sustain Talk (SS)	Avoids a focus on the reasons against changing or on maintaining the status quo.
Partnership (P)	Conveys an understanding that expertise and wisdom about change reside mostly within the client.
Empathy (E)	Understands or makes an effort to grasp the client's perspective and experience.

Behaviour counts (absolute measure)	
Giving Information (GI)	Gives information, educates, provides feedback, or expresses a professional opinion without persuading, advising, or warning.
Questions (Q)	Questions (open or closed).
Simple Reflection (SR)	Reflects a client's statement with little or no added meaning or emphasis.
Complex Reflection (CR)	Reflects a client's statement with added meaning or emphasis.
Affirm (AF)	States something positive about the client's strengths, efforts, intentions, or worth.
Emphasize Autonomy (EA)	Highlights a client's sense of control, freedom of choice, personal autonomy, ability, and obligation about change.
Confront (C)	Directly and unambiguously disagreeing, arguing, correcting, shaming, blaming, criticizing, labelling, warning, moralizing, ridiculing, or questioning a client's honesty.
Seek Collaboration (Seek)	Attempts to share power or acknowledge the expertise of a client.
Persuade with Permission (PwP)	Emphasis on collaboration or autonomy support while using direct influence.
Persuasion (Per)	Overt attempts to change a client's opinions, attitudes, or behaviors using tools such as logic, compelling arguments, self-disclosure, facts, biased information, advice, suggestions, tips, opinions, or solutions to problems.

Summary measures (fair threshold)	
Total MI non-adherent	MI non-adherent behaviours = total confront + persuade
Total MI adherent	MI adherent behaviours = total seek collaboration + affirm + emphasising autonomy
Reflections to question ratio (1:1)	Reflections to questions ratio = total reflections/total questions
Relational (3.5)	Relational = (partnership + empathy)/2
Technical [3]	Relational = (cultivating + softening)/2
% Complex reflections (40%)	Percent complex reflections = CR/(SR + CR) expressed as a %
  • Before commencing the trial all physiotherapists will attend a 2-h group discussion regarding the application of the MI principles with the pre-determined foci

After commencing the trial, physiotherapists will be evaluated for delivery of treatment by the following process:

• One of the 10 specific MI sessions (selected randomly excluding the initial session) will be audio recorded per participant and evaluated using the MITI on the middle 20 min of the session (excluding supervised exercise time)
• If the competency and proficiency threshold of “fair” is not met (Table 4), further training for the physiotherapists will be implemented followed by re-evaluation with the MITI
• If physiotherapists have not delivered trial treatment for more than 8 weeks further training will occur
• To examine potential contamination effects in the comparison group, each physiotherapist will provide an audio recording of one participant session receiving the comparison group treatment for scoring on the MITI
• Ten percent of the sessions coded will be randomly chosen for double coding for credibility. This percentage was determined a priori based on the expert recommendation of the MITI developers, with consideration of the principles of inter-rater reliability for observational data [43], and relevant factors related to participant numbers, physiotherapist numbers, and number of coders
• One of the authors (PO) will be available to the physiotherapists for ad hoc advice, and a group meeting between the authors (PO/JF) and physiotherapists will occur every 2 months to discuss cases for 60-90 min

The ability of participants to understand (treatment receipt) and perform in real life skills (treatment enactment) treatment related skills [44] will be evaluated via analysis of all participants’ (experimental and comparison group) goal sheets and the proportion of short, medium and long term goals achieved (treatment enactment).

### 1.11 Outcome measures

The primary outcome measures will comprise:

i) The Pain Self-Efficacy Questionnaire at 52-weeks;
ii) 0-10 numerical rating scale from the Brief Pain Inventory to measure how pain interferes with seven life domains (general activity, mood, walking ability, normal work, relations with others, sleep and enjoyment of life), with endpoint descriptors of “not interfered” and “completely interfered” at 52-weeks.

The primary outcomes will also be measured at secondary timepoints of 12 weeks and 26 weeks as described below.

Secondary outcome measures will comprise the remainder of self-administered questionnaires.

A set of self-administered questionnaires will be sent to participants by a study administrator and completed online by participants at baseline and at each of the follow-up timepoints (12 weeks, 26 weeks and 52 weeks post randomisation). All measures have demonstrated good reliability, responsiveness and evidence of validity in chronic pain populations. This battery of questionnaires covers the full range of outcome domains that have been recommended for measurement in all clinical trials on pain and is comprised of:

i. Brief Pain Inventory pain location, average pain intensity and pain interference scores over the previous week [45,46];
ii. The Pain Disability Questionnaire measuring activity limitation due to pain, with scores ranging from 0 to 150(48);
iii. Depending on the location of the main pain area, one of the Oswestry Disability Questionnaire [47], the Neck Disability Index [48], the Upper Extremity Functional Index [49] or the Lower Extremity Functional Index [50] measuring body region specific activity limitation due to pain;
iv. The Depression Anxiety and Stress Scales identify people at risk of clinical depression and anxiety [51,52];
v. The Pain Self-efficacy Questionnaire (PSEQ) measuring confidence to accomplish a range of behaviours/tasks despite pain [53];
vi. The Pain Catastrophising Scale (PCS) measuring thoughts and feelings related to their pain [54];
vii. The Short Form Örebro Musculoskeletal Screening Questionnaire as a prognostic screening tool [55];
viii. The Post Traumatic Stress Disorder Checklist (PCL-5) to screen for posttraumatic stress disorder [56,57];
ix. The Insomnia Severity Index which identifies severity of sleep continuity disturbance (e.g. initiating sleep, staying asleep, and early morning waking) [58,59];
x. The Pain related Beliefs and Attitudes about Sleep (PBAS) that assesses pain related cognitions about sleep [60];
xi. The Central Sensitisation Inventory [61];
xii. The Neuropathic Pain Questionnaire [62];
xiii. The Alcohol Use Disorders Identification Test-Consumption (AUDIT-C) that assesses alcohol misuse [63];
xiv. The Drug Use Disorders Identification Test–Consumption (DUDIT-C) that assesses drug misuse [64];
xv. The EuroQoL-5D-5L for measuring quality of life and allowing health state utilities to be calculated for the cost-effectiveness analysis;
xvi. A Participant Diary to track healthcare, medication, and radiological imaging utilisation, as well as number of work days missed;
xvii. Satisfaction with treatment measured on a 5-point Likert Scale;
xviii. Treatment Credibility Questionnaire [65] to determine the acceptability of each treatment approach;
xix. Participant adherence with treatment will be determined via the number of sessions attended, as well as a self-administered 0-10 rating scale at each follow-up: “On a scale of 0-10, how adherent have you been with following the advice and exercises recommended by your physiotherapist on average over the last month” (from “not at all” to “fully”) [66];
xx. Global rating of change will be measured on a 7-point scale ranging from “very much worse” to “very much better”.

Additional data will also be obtained at baseline only (gender, age, country of birth, height/weight, existence of a compensation claim, nature and history of pain, comorbidities).

Participants may be offered a $20-$50 (AUD) gift voucher if outcome measures are not completed at the primary outcome timepoint.

### 1.12 Sample size

An effect size (standardised mean difference) of 0.4 can be considered a reasonable minimum threshold of clinical importance between groups [67,68]. To provide 80% power to detect a between-group effect of this magnitude (alpha set at 0.05, 2-tailed hypothesis), 92 participants per group will be required (allowing for 10% loss to follow-up), and accounting for the higher power provided by incorporating baseline scores as a covariate [69] and assuming a correlation between baseline and follow-up scores of r = 0.4 supported by our previous trial [70]. On this basis 184 participants will be recruited. In a previous RCT completed by our research team, the 12-month attrition rate was 7.3% [70]. On this basis a total of 184 participants will be recruited.

### 1.13 Analysis

Data analysis will focus on detecting the between-group treatment effects at each follow-up. Continuous data will be analysed using linear mixed models (with the group × time interaction estimating the treatment effect), adjusting for baseline scores and the stratification variables (treatment centre and primary pain site). The inclusion of treatment centre as a random effect will account for the potential clustering of outcomes within treatment centres. Ordinal data will be analysed using the Mann Whitney *U* test.

To account for hypothesis testing on two primary outcomes, the family-wise Type I Error rate will be controlled at 0.05 using Holm's step-down procedure. The smaller p-value will first be compared with an alpha of 0.025, and if statistically significant the larger p-value will then be compared with an alpha of 0.05. The intervention will be considered effective overall if at least one primary outcome is statistically significant under this procedure. Conclusions will be reported separately for BPI pain interference and pain self-efficacy.

All data will be analysed on an intention to treat basis, in that all participants will be analysed in the treatment group to which they are initially allocated regardless of their compliance with that treatment. All participants who withdraw from treatment for any reason will continue to be contacted for follow-up assessments and informed that their data are still required. Missing data will be primarily handled via maximum likelihood estimation within the linear mixed models. As a secondary sensitivity analysis, missing data will be modelled via multiple imputation.

A within-trial cost-effectiveness analysis will be undertaken. The evaluation will be from the healthcare perspective, with work absence data to be considered separately. Validated algorithms will be used to generate health state utilities from the EQ-5D-5L. Cost data will be calculated from participant diaries (see outcome assessment). Differences in incremental health outcomes and incremental costs between the groups will be calculated using linear mixed models. The incremental cost effectiveness ratio (ICER) will then be derived, to indicate the cost to gain one additional quality adjusted life year. To assess the uncertainty around the ICER, non-parametric bootstrapping will be applied using 5000 randomly sampled data sets. Each of the 5000 bootstrapped ICERs will be plotted on the cost-effectiveness plane. A cost-effectiveness acceptability curve will then be generated to determine the probability that any of the treatment groups are cost-effective at different willingness to pay thresholds.

### 1.14 Mediators and treatment effect modifiers

Treatment effect modifier analyses will determine participant characteristics at baseline associated with either a larger or smaller treatment effect from PM + MI relative to PM alone. The hypotheses to be tested will be that the greatest benefit from PM + MI relative to PM alone will be in participants with higher scores on one or more of the Short Form Örebro Musculoskeletal Screening Questionnaire and/or the Pain Disability Questionnaire and/or lower scores on the Pain Self-efficacy Questionnaire at baseline.

Mediator analyses will determine potential mechanisms of action for the motivational interviewing intervention evaluating:

1) Whether self-efficacy mediates the effect of PM + MI versus PM treatment on pain interference
2) Whether practitioner motivational interviewing behaviours as measured by the MITI mediate the effect of PM + MI versus PM treatment on self-efficacy and pain interference

In addition to the pre-planned analysis, a post-hoc data-driven approach will be employed to identify new hypotheses for future verification and exploration of potential mechanisms.

## 2 Discussion

This trial will evaluate the effectiveness of adjunctive MI for people with nociplastic dominant pain undergoing multi-disciplinary pain management on improving pain self-efficacy and pain interference at 52-weeks in people with nociplastic dominant chronic pain. Given the strong treatment fidelity program and the potential for MI to mediate behaviour change and self-efficacy, the results have the potential to improve the size of effects observed to date in RCTs on chronic pain to date. This is particularly relevant for people with proposed nociplastic dominant chronic pain that may be more resistant to standard pain management.

Several limitations may affect interpretation of the trial results. The method for determination of dominant pain type has only been partially validated and this eligibility criterion may restrict generalisability. Treating physiotherapists cannot be blinded, and participants may recognise aspects of their treatment despite limited disclosure of the hypothesis.

If PM + MI improves outcomes, the results would support consideration of this MI as an adjunctive treatment to multi-disciplinary pain management provided the described treatment fidelity methods were adhered to. The results on pain interference and cost-effectiveness have the potential to significantly reduce the burden of chronic pain across the world.

## CRediT authorship contribution statement

**Jon Ford:** Writing – review & editing, Writing – original draft, Visualization, Validation, Supervision, Software, Resources, Project administration, Methodology, Investigation, Funding acquisition, Formal analysis, Data curation, Conceptualization. **Paul O'Halloran:** Writing – review & editing, Writing – original draft, Visualization, Validation, Supervision, Methodology, Investigation, Data curation, Conceptualization. **Nicholas F. Taylor:** Writing – review & editing, Writing – original draft, Visualization, Validation, Supervision, Methodology, Investigation, Data curation, Conceptualization. **Shane Pritchard:** Writing – review & editing, Writing – original draft, Visualization, Validation, Supervision, Software, Resources, Project administration, Methodology, Investigation, Formal analysis, Data curation. **Andrew Hahne:** Writing – review & editing, Writing – original draft, Visualization, Validation, Supervision, Software, Resources, Project administration, Methodology, Investigation, Funding acquisition, Formal analysis, Data curation, Conceptualization.

## Ethics declaration

Written informed consent to take part in the study and to publish the article has been obtained from all participants or their legal representatives. The privacy rights of participants have been observed.

This study was performed in compliance with relevant laws, regulatory frameworks and guidelines where the research took place. This study was approved by the La Trobe University Human Ethics Committee. (Approval No. #HEC21026)

The results of this clinical trial and any associated work have been posted in a registry. This clinical trial was registered with number ACTRN12622000829718 (Australia & New Zealand Clinical Trials Registry).

## Funding sources

Advance Healthcare provide facilities, personnel and resources to allow treatment of trial participants free of charge.

## Declaration of competing interest

The authors declare the following financial interests/personal relationships which may be considered as potential competing interests:Jon Ford reports financial support and administrative support were provided by Advance Healthcare. Jon Ford reports a relationship with Advance Healthcare that includes: board membership, employment, and equity or stocks. If there are other authors, they declare that they have no known competing financial interests or personal relationships that could have appeared to influence the work reported in this paper.

## Data Availability

No data was used for the research described in the article.
